# Evaluation of *Macaca radiata* as a non-human primate model of Dengue virus infection

**DOI:** 10.1038/s41598-018-21582-9

**Published:** 2018-02-21

**Authors:** Fumihiro Kato, Yuki Ishida, Akihiko Kawakami, Tomohiko Takasaki, Masayuki Saijo, Tomoyuki Miura, Takayuki Hishiki

**Affiliations:** 1 Laboratory of Primate Model, Institute for Frontier Life and Medical Sciences, Kyoto University, Kyoto, Japan; 2 Department of Virology 1, National Institute of Infectious Diseases, Tokyo, Japan; 3 Present Address: Department of Virology 1, National Institute of Infectious Diseases, Tokyo, Japan; 4 Present Address: Kanagawa Prefectural Institute of Public Health, Kanagawa, Japan; 5 Present Address: Department of Microbiology and Cell Biology, Tokyo Metropolitan Institute of Medical Science, Tokyo, Japan

## Abstract

Dengue virus (DENV) causes a wide range of illnesses in humans, including dengue fever and dengue haemorrhagic fever. Current animal models of DENV infection are limited for understanding infectious diseases in humans. Bonnet monkeys (*Macaca radiata*), a type of Old World monkey, have been used to study experimental and natural infections by flaviviruses, but Old World monkeys have not yet been used as DENV infection models. In this study, the replication levels of several DENV strains were evaluated using peripheral blood mononuclear cells. Our findings indicated that DENV-4 09-48 strain, isolated from a traveller returning from India in 2009, was a highly replicative virus. Three bonnet monkeys were infected with 09-48 strain and antibody responses were assessed. DENV nonstructural protein 1 antigen was detected and high viraemia was observed. These results indicated that bonnet monkeys and 09-48 strain could be used as a reliable primate model for the study of DENV.

## Introduction

Dengue virus (DENV), which is transmitted to humans by *Aedes* mosquitoes, is the aetiological agent of dengue fever (DF) and dengue haemorrhagic fever (DHF), a self-limited febrile illness^1^. DF is relatively mild, but DHF leads to life-threatening dengue shock syndrome (DSS); the mortality rates of DHF and DSS in untreated cases exceed 20%, but decrease to less than 1% with proper medical care^2^. DENV infects 50–100 million humans annually in tropical and subtropical regions, posing a considerable threat to public health in over 100 countries^3^.

Despite great concern worldwide, current strategies for prevention and treatment are not sufficient, partly owing to a lack of animal models reflecting dengue clinical symptoms. Experimental animal models have been established for various viruses, such as retroviruses and influenza virus; these models are indispensable for evaluating the antiviral activity and side effects of potential treatments and for antiviral drug development. Moreover, animal models can be used to determine the dynamics and pathological mechanisms of viruses, which can facilitate the development of appropriate antiviral strategies^4–8^.

Some mouse and non-human primate (NHP) models have been developed for the study of DENV^9–11^. However, these animal models have limits for pathological and virologic analyses and are genetically differentiated from humans^12^. In an immunocompromised mouse model in which interferon-α, -β and -γ receptors were knocked out, highly pathogenic DENV infection was observed. However, an immunological response was not observed using immunocompromised mice and mouse models are not sufficient for studies of human disease^13^. The common marmoset has also been used as an animal model of DENV and high viraemia and immune responses corresponding to those in humans have been observed^10,14^. However, the common marmoset is a New World monkey and Old World monkeys may be more representative of humans. New world monkeys differ from Old World monkeys with respect to immune responses, including differences in major histocompatibility complex alleles and antibody cross reactivity. Indeed, Old World monkeys are more similar to humans than are other available animal models^15–17^. Old World monkeys have been used as DENV infection models^18–21^. However, experimental inoculation results in low viraemia levels and only mild symptoms^22^. Therefore, the establishment of new animal models that are genetically closely related to humans and have reproducible symptoms of human DENV infection would provide a powerful tool for understanding the mechanisms of DENV-induced pathogenesis and for the development of antiviral drugs and vaccines.

Bonnet monkeys (*M. radiata*) are indigenous to Southern India, where DENV circulates widely. In previous studies, Kyasanur forest disease virus (KFDV), tick-borne encephalitis virus (TBEV), Japanese encephalitis virus (JEV) and West Nile virus (WNV) have been inoculated into bonnet monkeys, resulting in high viraemia^23–25^. Furthermore, neutralisation antibodies have been detected after natural infection in bonnet monkeys^26^. However, the experimental inoculation of DENV using bonnet monkeys has not yet been reported.

Some reports have shown that sylvatic DENV is transmitted between NHPs and mosquitoes in the sylvatic environment. It is phylogenetically distinct from urban DENV circulating in humans^27–31^. It is thought that sylvatic DENV is an effective candidate challenge virus for the development of an NHP model.

In this study, to develop a novel NHP model for DENV infection, we evaluated the replication efficiency of five DENV isolates using peripheral blood mononuclear cells (PBMCs) derived from *M. radiata*. The most replicative DENV, 09-48 strain, was used to infect bonnet monkeys and a biological analysis was performed using plasma samples. We also performed a phylogenetic analysis to determine the relationships between this highly replicative strain and other isolates.

## Results

### Comparison of the replication of clinically isolated DENVs in PBMCs of *M. radiata*

**Figure 1:**
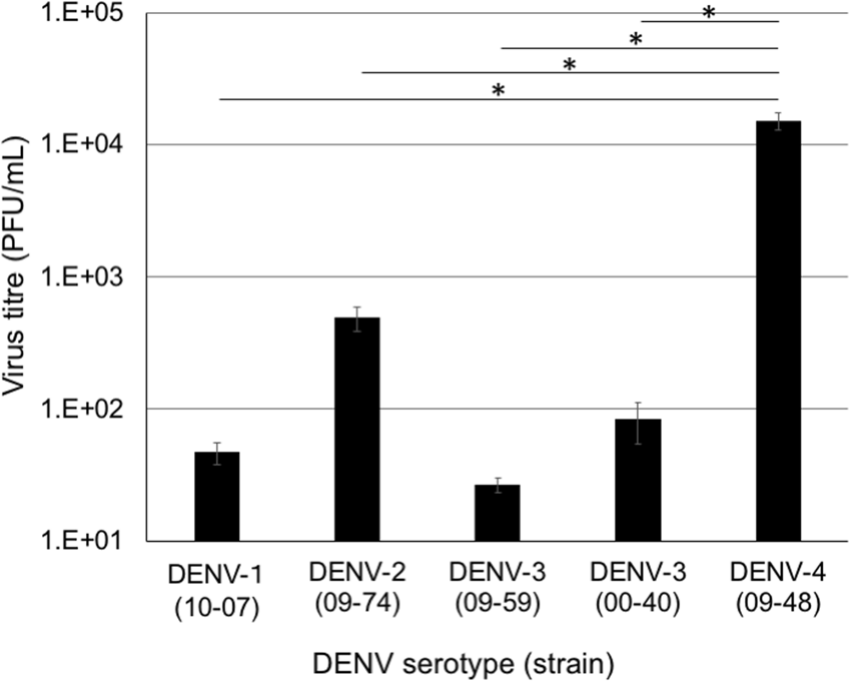
DENV replication in *M. radiata* PBMCs. PBMCs derived from *M. radiata* were infected with DENV at an MOI of 0.1. At 2 dpi, the viral titres in culture supernatants were determined by plaque assays using BHK-21 cells and three independent tests were performed. Results were compared using the Student’s *t*-test against DENV-4 and P < 0.05 (*) was considered statistically significant.

**Figure 2:**
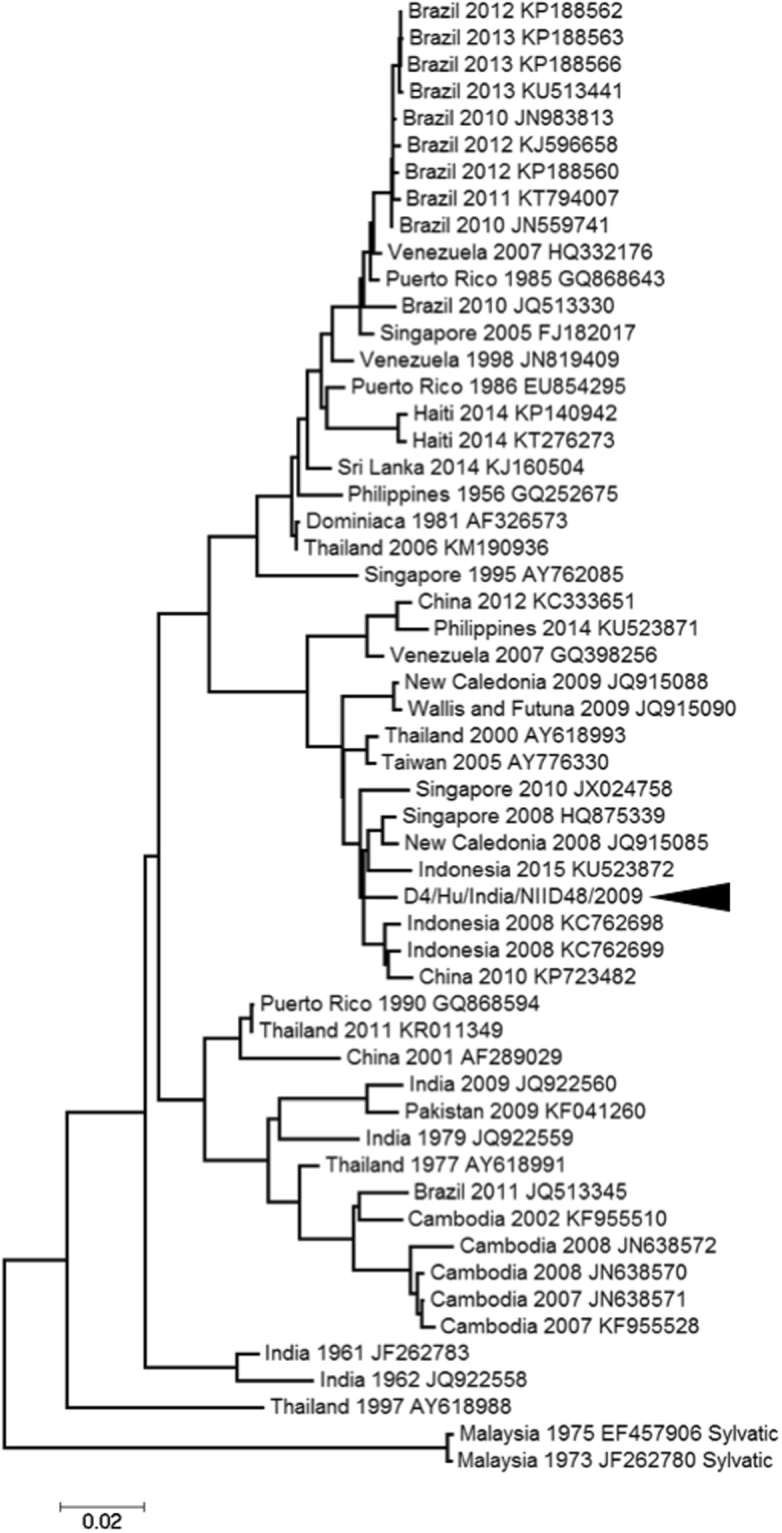
Phylogenetic analysis of DENV 09-48 strain. A phylogenetic tree was constructed using the maximum likelihood method with the DENV-4 polyprotein coding region. The DENV-4 09-48 strain is indicated by the black triangle. The country and year of isolation as well as the accession number are indicated.

To investigate whether clinically isolated DENV strains were capable of efficient replication in primary cells derived from *M. radiata*, equal MOIs of DENV strains were infected and titres of progeny viruses from PBMCs at 2 dpi were measured by plaque assays. The viral titres of DENV-1 (10-07), DENV-2 (09-74), DENV-3 (09-59), DENV-3 (00-40) and DENV-4 (09-48) were 4.7 × 10^1^, 4.9 × 10^2^, 2.7 × 10^1^, 8.3 × 10^1^ and 1.5 × 10^4^ PFU/mL, respectively (Fig. 1). DENV-4 (09-48) showed significantly higher progeny virus titres than those of other isolates, suggesting that DENV-4 (09-48) has potential applications as a challenge virus candidate. Furthermore, the sequence of the 09-48 strain was determined (accession number: LC069810) and analysed using phylogenetic methods (Fig. 2). DENV-4 (09-48 strain) belonged to a cluster of strains isolated from Indonesia in 2004 and 2008 and was divergent from sylvatic strains.

### Infection of *M. radiata* with DENV-4

**Figure 3:**
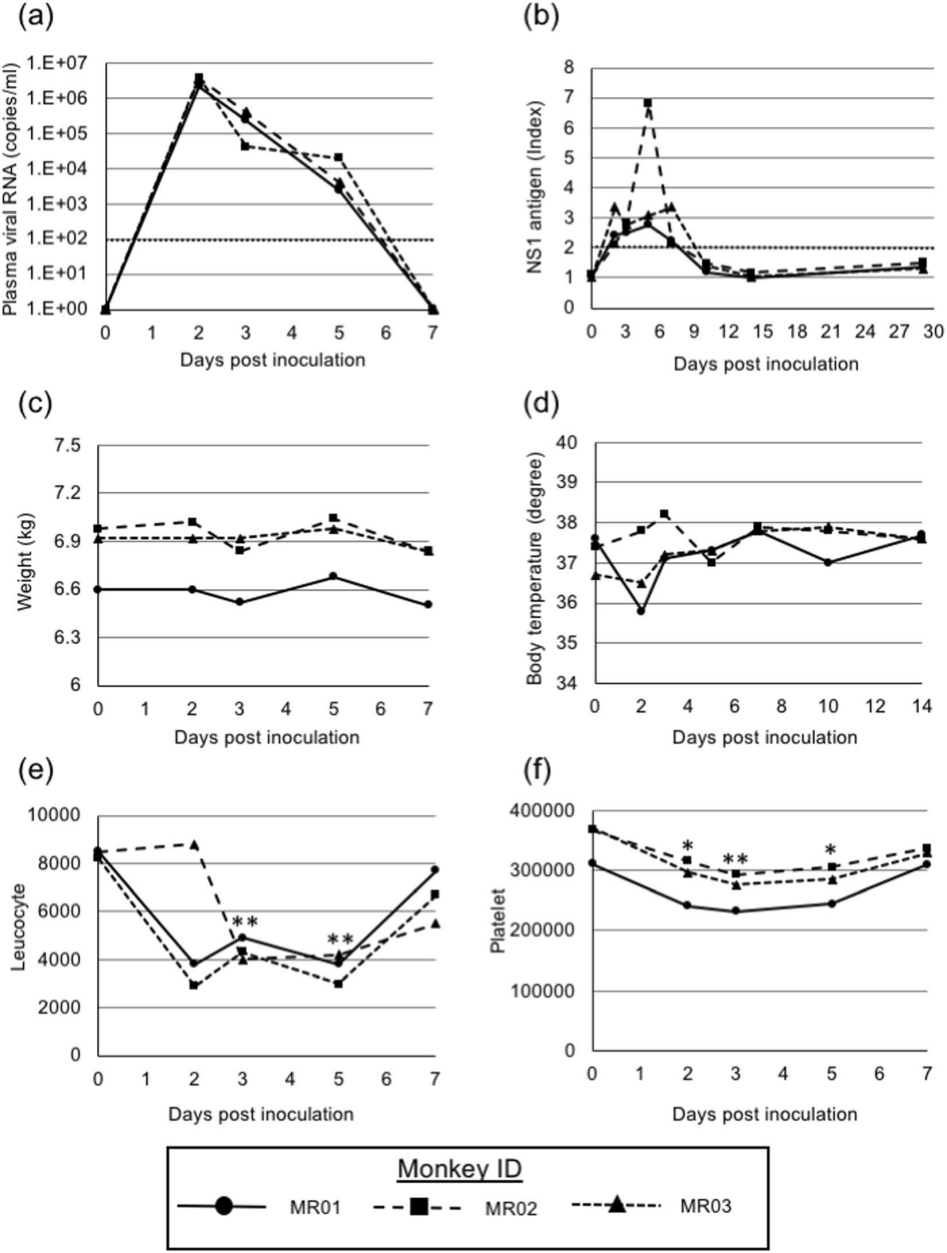
Detection of viral RNA and NS1 protein antigen and profiles of leukocytes and platelets. (**a**) Plasma samples were collected on the indicated days. The amount of viral RNA in the plasma of three individual *M. radiata* was determined by qRT-PCR. The dotted line indicates the detection limit (<100 copies/mL). Growth curves of *M. radiata* were evaluated using two-way ANOVA. (**b**) The level of the DENV NS1 antigen was analyzed by ELISA. The dotted line indicates the criterion for positive or negative results. Index values of greater than 2 were considered positive. (**c**) Body weights of three *M. radiata*. (**d**) Rectal temperatures of three *M. radiata*. (**e** and **f**) Blood samples were collected on the indicated days. The absolute number of leukocytes (**e**) and platelets (**f**) in blood were determined using an automated haematology analyser. Results were compared using the paired *t*-test against pre-inoculation levels; P < 0.05 (*) and P < 0.01 (**) were considered statistically significant.

Three *M. radiata* were inoculated intravenously with 1 × 10^6^ PFU of DENV-4 (09-48 strain). The collection of plasma samples, measurement of rectal temperatures and observations of the clinical presentation of monkeys were performed before inoculation and at 2, 3, 5, 7, 10, 14 and 29 dpi. Viraemia is a major clinical manifestation of DENV infection. The amount of viral RNA was analysed in plasma by quantitative RT-PCR at various time points. The viral load peaked at 2 dpi, reaching 2.2–4.0 × 10^6^ copies/mL, followed by a gradual decrease to below the limit of detection and there were no significant differences among *M. radiata* individuals (Fig. 3a). Furthermore, the NS1 antigen, as examined by ELISA, was detected in all plasma samples derived from DENV-4-infected *M. radiata* (Fig. 3b). These results suggested that DENV-4 propagated in all *M. radiata*. However, no clinical symptoms, e.g. haemorrhage or weight loss, were observed and rectal temperatures were not substantially altered at any time point (Fig. 3c and d).

### Profiles of leukocytes and platelets in blood

Collected blood samples were analysed to determine their cell properties using an automated haematology analyser. Both leukocytes and platelets were slightly decreased at 2, 3, 5 and 7 dpi (Fig. 3e and f).

### Analysis of antibody responses

**Figure 4:**
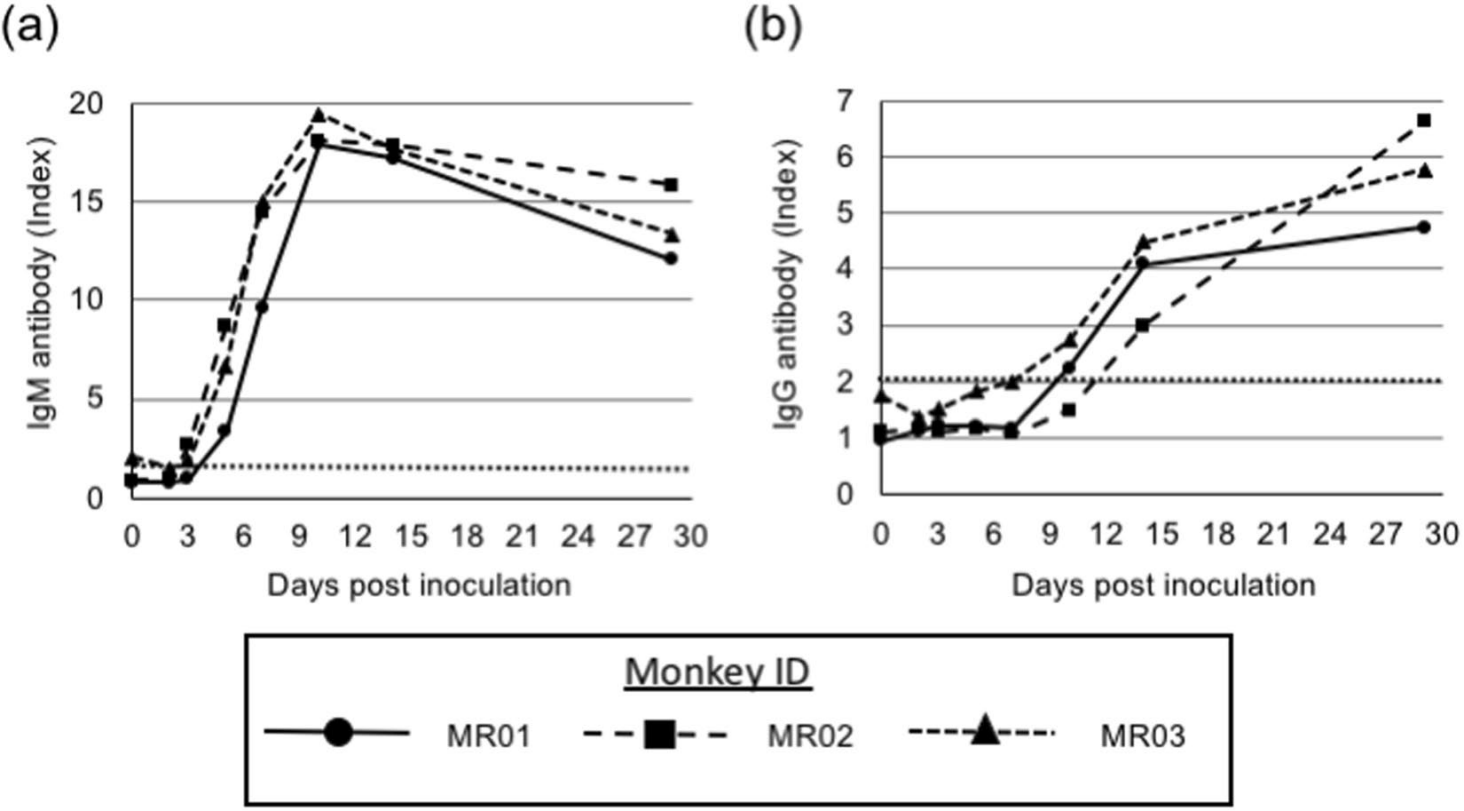
Analysis of DENV-specific IgM and IgG antibodies. Plasma samples were collected on the indicated days. The levels of DENV-specific IgM (**a**) and IgG (**b**) antibodies were detected by ELISA. MR01 is indicated as circles/solid line, MR02 is indicated as squares/broad-dashed line and MR03 is indicated as triangles/narrow-dashed line in all figures. The dotted line indicates the criterion for positive or negative results. Index values of greater than 2 were considered positive.

IgG and IgM antibodies against DENV antigens were assessed by ELISA. In cases of DENV infection, IgM antibodies can be detected immediately and IgG antibodies subsequently increase. We detected IgM antibodies after 3 or 5 dpi, with a peak at 11 dpi. We detected IgG antibodies at 11 or 14 dpi and their levels increased thereafter (Fig. 4a and b). These results indicated that DENV-4 propagated and induced specific IgM and IgG antibodies in *M. radiata*.

### Neutralisation antibodies

Neutralisation antibodies were measured by plaque reduction assays. Neutralisation antibodies were observed at 5 or 7 dpi. At 29 dpi, neutralisation antibody titres for all three monkeys were increased to over 1:160 (Table 1).

**Table 1:** Neutralising antibody titres targeting DENV-4.

Days postinoculation	2	3	5	7	9	11	14	29
MR01	<1:10	<1:10	<1:10	1:40	1:40	1:160	1:160	1:160
MR02	<1:10	<1:10	<1:10	1:40	1:40	1:40	1:160	1:160
MR03	<1:10	<1:10	1:10	1:40	1:40	1:160	1:80	1:160

## Discussion

In this study, five clinically isolated DENV strains were evaluated to determine viral replication in PBMCs derived from bonnet monkeys. DENV-4 09-48 strain was isolated from a traveller returning to Japan from India and showed the most efficient replication in PBMCs, suggesting that this strain may be a suitable challenge virus in bonnet monkeys. An evaluation of growth kinetics in PBMCs before *in vivo* analyses may provide insights into the appropriate growth level *in vivo* for this challenge virus.

Sylvatic DENV, which is distinguished from urban DENV circulating in humans, has been evaluated in various studies^27–31^. Sylvatic DENV cycles between NHPs and mosquitoes in the sylvatic environment and studies have evaluated its genome and isolation. If sylvatic DENV is acclimatised to monkeys, it may be an effective candidate challenge virus for the development of an NHP model. The sequence of the 09-48 strain indicated that it belongs to the urban DENV cluster and is divergent from the sylvatic DENV cluster. Some sequence differences may affect viral replication in PBMCs; further analyses are needed.

In this study, 09-48 strain was intravenously injected into *M. radiata*. The results should be interpreted with caution because intravenous inoculation is a different route compared with natural DENV infection. In cases of experimental infection in Old World monkeys, viraemia levels are typically very low. However, following the infection of *M. radiata* with DENV-4 09-48 strain, viraemia levels were very high^19,32,33^, even soon after infection. The peak viral titre was observed within 2 dpi. The NS1 antigen, which is a product of viral infection and replication, was also observed, as evidence of DENV replication in *M. radiata in vivo* and inoculated DENV stock reached a titre of 5.0 × 10^8^ copies/mL. The monkeys used in this study weighed 6–7 kg and the total serum quantity was approximately 250 mL. After inoculation, there was an estimated 2.0 × 10^6^ copies/mL and viral titres at 2 dpi were similar to or greater than the inoculation titre, suggesting that much of the detected viral genome was derived from *in vivo* replication. Additionally, *M. radiata* showed lower viraemia levels than those of marmosets, but higher viraemia levels than those of many other Old World monkeys.

In typical DENV infection in humans, IgM antibodies are detected 3–5 days after onset and IgG antibodies are detected 10–14 days after onset^34,35^. Human-like antibody responses against DENV infection are important for the establishment of animal infection models. However, in humans or marmosets, infection with DENV resulted in higher IgG or IgM antibody responses compared with those in *M. radiata*. Notably, *M. radiata* could be applied for antiviral drug or antibody medical treatment for DENV infection owing to the similarities in genetic backgrounds between *M. radiata* and humans.

The platelet and leukocyte counts were slightly and transiently decreased by about 24% and 54%, respectively, similar to the counts in previous marmoset experiments^14^. Furthermore, the recovery times of leukocytes and platelets were similar to those of humans following DENV infection^36^. In this study, the body weight and the amount of blood collected for *M. radiata* were similar to those for other experiments using Old World monkeys^6,37^, suggesting that blood collection did not affect the hemogram properties caused by DENV-4 infection.

Neutralisation antibodies were also detected by plaque reduction assays in this study. In comparison with DENV infection results in primate models, such as marmosets and rhesus macaques, neutralisation titres in DENV-4-infected *M. radiata* were similar^19,38^. Compared with human infection, neutralisation antibody levels were significantly lower; however, the timing of the increases in IgM, IgG and neutralisation antibodies was similar, suggesting that this model was useful for the evaluation of neutralisation levels induced by vaccines or viral infection.

A previous report has shown that clinical symptoms, such as rash, were induced by DENV infection in NHPs^19^. In this study, although clear clinical symptoms were not observed, a rash-like bruise was present in MR02 (data not shown). However, this bruise was not analysed using pathological methods, such as immunostaining. Furthermore, viraemia and other responses in MR02 were not different from those in MR01 and MR03. Therefore, this bruise may not a result of DENV infection. In animal model inoculation studies, observations of human-like clinical symptoms are essential. However, in this study, these symptoms of DENV infection were not found and further analyses are needed to explain this result.

In conclusion, *M. radiata* showed high viraemia levels and human-like antibody responses after infection with DENV-4 09-48 strain. The establishment of new animal models, particularly Old World monkeys, is needed to understand the molecular mechanisms of DENV infection and to develop effective antiviral drugs and vaccines. Further studies of *M. radiata* and other Old World monkeys as DENV infection models are needed.

## Methods

### Cells and viruses

BHK-21 and C6/36 cells were maintained in Eagle’s Minimum Essential Medium containing 10% foetal bovine serum (FBS) and Non-Essential Amino Acid. PBMCs were prepared from ethylenediaminetetraacetic acid-treated whole blood in lymphocyte separation medium. PBMCs were resuspended in R-10 medium supplemented with 40 μg/mL gentamicin, 50 μM 2-mercaptoethanol and 25 μg/mL concanavalin A and cultured for 16–20 h at 37 °C. Before virus infection, the cells were cultured for an additional 2 days in R-10 medium containing 40 μg/mL gentamicin, 50 μM 2-mercaptoethanol and 100 IU/mL recombinant human interleukin-2. DENVs were propagated with C6/36 cells and the titres were measured by plaque forming unit (PFU) assays using BHK-21 cells. DENV-1 10-07 was isolated in 2010 from a traveller returning from Indonesia; DENV-2 09-74 was isolated in 2009 from a traveller returning from India; DENV-3 09-59 and 00-40 were isolated in 2009 from a traveller returning from Papua New Guinea and in 2000 from a traveller returning from Thailand, respectively; and DENV-4 09-48 was isolated in 2009 from a traveller returning from India^39,40^.

### Analysis of viral replication in PBMC of *M. radiata*

Growth properties of five clinically isolated strains were investigated in PBMCs derived from three bonnet monkeys and 1 × 10^6^ cells for each type of PBMC were seeded on 24-well plates and infected with DENV at a multiplicity of infection (MOI) of 0.1. At 2 days after infection, supernatants were harvested and viral titres were measured by plaque assays, as described previously^41^.

### Infection of *M. radiata* with DENV-4

*M. radiata* was used in accordance with the institutional regulations approved by the Committee for Experimental Use of Nonhuman Primates of the Institutes for Virus Research, Kyoto University, Kyoto, Japan. Briefly, 1 × 10^6^ PFU of DENV-4 09-48 strain was intravenously inoculated to three *M. radiata* (6–7 kg in body weight) after anaesthetisation with ketamine hydrochloride (Daiichi-Sankyo, Tokyo, Japan). Blood samples were collected and rectal temperatures were measured at 2, 3, 5, 7, 10, 14 and 29 days postinoculation (dpi). Collected blood samples were separated into serum and cells by centrifugation.

### Profiles of leukocytes and platelets in blood

The numbers of leukocytes and platelets in blood were measured using a multiple automatic blood cell counting device (KX-21; Sysmex, Kobe, Japan).

### IgG and IgM antibodies and nonstructural protein 1 (NS1) antigen detection

Detection of IgG and IgM antibodies and anti-NS1 antigen in the serum was performed using enzyme-linked immunosorbent assays (ELISAs) with Dengue Virus IgG DxSelect (Focus Diagnostics, Cypress, CA, USA), Dengue Virus IgM Capture DxSelect (Focus Diagnostics) and a PLATELIA DENGUE NS1 AG Kit (Bio-Rad, Hercules, CA, USA). Values of greater than 2 were considered positive according to the manufacturer’s protocol.

### Plaque reduction assay

Sera were diluted with FBS by 10–160-fold and mixed with 50 PFU of virus at a ratio of 1:1. The mixed samples were then incubated at 37 °C for 30 min. BHK cells were seeded in 12-well plates (5 × 10^5^ cells/well) and inoculated with samples for 1 h. After inoculation, E-MEM containing 2% FBS and 1% methyl cellulose was overlaid and incubated for 5–6 days. Fixation and staining were applied as described for plaque assays. Samples with 50% fewer plaques than those of the negative control were considered positive.

### Real-time RT-PCR

Viral RNA was extracted from plasma using a High Pure Viral RNA Kit (Roche, Basel, Switzerland) according to the manufacturer’s instructions. A quantitative real-time RT-PCR analysis of the envelope region of the DENV-4 genome was performed using RNA-direct Real-time PCR Master Mix (Toyobo, Osaka, Japan) and a StepOnePlus System (Applied Biosystems, Foster City, CA, USA). DENV-4-specific primer-probe sets (D4Ten711s forward: GGTGACRTTYAARGTHCCTCAT, D4Ten786c reverse; WGARTGCATRGCTCCYTCCTG and TaqMan probe: D4Ten734p probe, CCAAGAGACAGGATGTGACAGTGCTRGGATC), developed by Ito *et al*.^39^, were used in this study. Note that in the primer sequences, W indicates a mix of A and T, R indicates A and G, Y indicates C and T and H indicates A, T and C. The real-time RT-PCR conditions were as follows: denaturation (90 °C for 30 s), reverse transcription (61 °C for 20 min), denaturation (95 °C for 1 min) and 40 cycles of amplification and quantification (95 °C for 15 s, 57 °C for 1 min). The copy number was determined by the relative quantification method using the synthesized RNA template.

### Determination of DENV-4 09-48 strain sequences

DENV RNA was isolated using a High Pure viral RNA Kit (Roche) according to the manufacturer’s instructions. cDNA was synthesised from viral RNA and three fragments were amplified by polymerase chain reaction (PCR) using Q5 High-Fidelity 2× Master Mix (NEB, Ipswich, MA, USA) and the following primers (fragment1: D4.001 f, AGTTGTTAGTCTGTCTGGACCG; D4.4620r, TCCCAAACAACCCTCTTTGCAT; fragment2: D4.4210 f, TCCCTTTAGCTGGCCCAATGGT; D4.9427r, TGGCGGATGAGTTGTACTTCCAT; fragment3: D4.8976 f, CGAGCAATCTGGTATATGTGG; D4.10649r, AGAACCTGTTGGATCAACAACAC). Purified PCR products were sequenced using primers (Supplementary Table S1) and analysed using a Big Dye Terminator v3.1 Kit and an ABI 3500 sequencer (Applied Biosystems).

### Phylogenetic analysis

A phylogenetic tree using the full sequence of the 09-48 strain was constructed by the maximum-likelihood method^42^.

## Electronic supplementary material

Supplementary Table
