# Supplementary material for: Evaluation of Macaca radiata as a non-human primate model of Dengue virus infection
Source: Sci Rep. 2018 Feb 21;8:3421. doi: 10.1038/s41598-018-21582-9 (PMC5821881; doi:10.1038/s41598-018-21582-9)
Supplement: Supplementary file 1 — Supplementary Table [file 41598_2018_21582_MOESM1_ESM.docx]

| Primer name | Sequence |
| --- | --- |
| D4.001f | AGTTGTTAGTCTGTGTGGACCG |
| D4.622f | CCGAAGACATTGATTGCTGGTG |
| D4.1221f | GACATGGTAGATAGAGGGTGG |
| D4.1821f | AAGGGAATGTCATACACGATGTG |
| D4.2409f | TTCACAGTCCATGCAGATATGG |
| D4.3033f | AGCTCAAAAAACCAGACCTGGCA |
| D4.3612f | TTAGGAGGTCTTACATGGATGGA |
| D4.4210f | TCCCTTTAGCTGGCCCAATGGT |
| D4.4770f | TGGAGGCTCGGAGACAAATGGGA |
| D4.5421f | ATCTCAACCAGGGTGGAAATGG |
| D4.5968f | ATGAAGATCATGCCCACTGGAC |
| D4.6576f | GGCATCTTCCTATTCTTCATGCA |
| D4.7224f | AGGACAGCTGCTGGGATCATGA |
| D4.7816f | GAGGATGGTCGTACTATATGGC |
| D4.8418f | GAAGAGCACAAAGAAACTTGGCA |
| D4.8976f | CGAGCAATCTGGTATATGTGG |
| D4.9626f | GAGGAAAGACATTCCGCAATGG |
| D4.10203f | GTGGATTACATGCCAGCCATGA |
| D4.455r | CGTTGACAAGTGAAACGCCAT |
| D4.1018r | TGTTCTAGCACCAGATCGACCCA |
| D4.1651r | ACCATTCTCTCTTTGTGATTCCA |
| D4.2194r | GCTGTTTCACCTAGAATGGCCAT |
| D4.2893r | TCTTCCACCTCAAGAAAGTTCCA |
| D4.3376r | ATCGTGCAGGAGCGACAGCACCA |
| D4.3985r | GCCATTATGGTCCTCCAAGCCAT |
| D4.4620r | T CCCAAACAACCCTCTTTGCAT |
| D4.5234r | TCCACGTAGCGCTTCTTCCAT |
| D4.5749r | TCTGTGGTAACCACAAAATCCCA |
| D4.6328r | GCGTAAACACGTGCATCTAACCA |
| D4.6952r | GTAGCTACTGCATAAAGCGTCCA |
| D4.7620r | CGAGTTTAGCTGTCTCTTCCA |
| D4.8234r | CACACCTGACACCCAATACAT |
| D4.8720r | CCCGAGGAGAGTCCACAGCCA |
| D4.9427r | TGGCGGATGAGTTGTACTTCCAT |
| D4.10084r | CCTAGGTAAGGTATGTCTTCCCA |
| D4.10649r | AGAACCTGTTGGATCAACAACAC |

Supplementary Table. List of primer sequences.
